# Underpinning the Development of Seaweed Biotechnology: Cryopreservation of Brown Algae (*Saccharina latissima*) Gametophytes

**DOI:** 10.1089/bio.2018.0147

**Published:** 2019-10-10

**Authors:** Wouter Visch, Cecilia Rad-Menéndez, Göran M. Nylund, Henrik Pavia, Matthew J. Ryan, John Day

**Affiliations:** ^1^ Department of Marine Sciences, Tjärnö Marine Laboratory, University of Gothenburg, Strömstad, Sweden.; ^2^ Culture Collection of Algae and Protozoa, Scottish Association for Marine Science, Scottish Marine Institute, Oban, United Kingdom.; ^3^ CABI, Bakeham Lane, Egham, Surrey, United Kingdom.

**Keywords:** aquaculture, brown algae, cryopreservation, gametophyte, kelp, *Saccharina latissima*

## Abstract

Sugar kelp (*Saccharina latissima*) is an economically important species, and natural populations provide diverse and productive habitats as well as important ecosystem services. For seaweed aquaculture to be successful in newly emerging industry in Europe and other Western countries, it will have to develop sustainable production management strategies. A key feature in this process is the capacity to conserve genetic diversity for breeding programs aimed at developing seed stock for onward cultivation, as well as in the management of wild populations, as potentially interesting genetic resources are predicted to disappear due to climate change. In this study, the cryopreservation of male and female gametophytes (haploid life stage) of *S. latissima* by different combinations of two-step cooling methods and cryoprotectants was explored. We report here that cryopreservation constitutes an attractive option for the long-term preservation of *S. latissima* gametophytes, with viable cells in all treatment combinations. The highest viabilities for both male and female gametophytes were found using controlled-rate cooling methods combined with dimethyl sulfoxide 10% (v/v). Morphological normal sporophytes were observed to develop from cryopreserved vegetative gametophytic cells, independent of treatment. This indicates that cryopreservation is a useful preservation method for male and female *S. latissima* gametophytes.

## Introduction

In cold-temperate regions, kelp forests represent important biological elements of coastal ecosystem, providing diverse and productive habitats as well as important ecosystem services.^1^ Furthermore, seaweeds (macroalgae) are economically important, with a global production of 30 Mton and a net worth of US$5.6 billion, of which kelps represent ∼34% of the total biomass.^2^ Seaweed aquaculture is the fastest growing sector of global aquaculture,^2^ but a relatively new industry in Europe and other Western countries. For seaweed aquaculture to be a successful emerging industry in these regions, it will have to develop sustainable production management strategies. A key feature in this process is the capacity to conserve genetic diversity for selective breeding programs to produce seed stock for onward cultivation and specific crossings.^3^ Conservation of genetic diversity is also an important prerequisite for the future management of wild populations as genetic resources and unique haplotypes have been predicted to disappear due to climate change.^4^ Ideally, methodologies to achieve these goals should employ a cost-effective approach that guarantees stable and long-term storage of the algae.

**FIG. 1.**
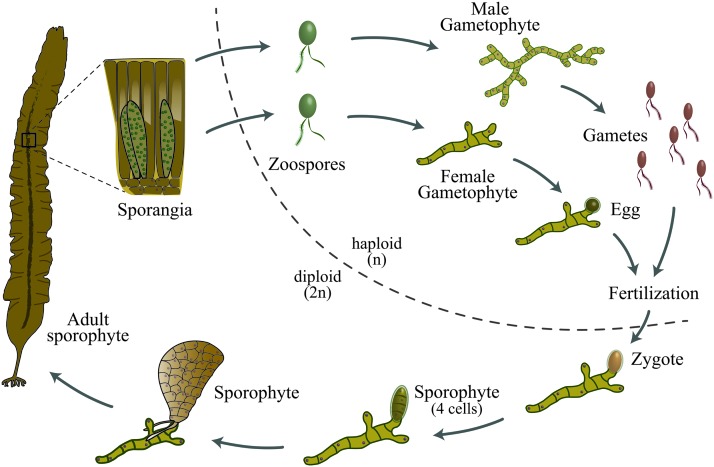
Life cycle of kelp (e.g., *Saccharina latissima*). During meiosis, zoospores (*n*) are formed in sporangia by a large multicellular sporophyte (2*n*). The spores settle onto the seafloor and develop into male and female gametophytes (*n*). Sterile gametophytes can be clonally propagated, and used as seed stock for further breeding and cultivation. Male and female gametophyte form antheridia that produce sperm and oogonia that produce eggs, respectively. The sperm fertilizes the egg, and a zygote is formed that develops into a sporophyte (2*n*). Color images are available online.

In the northern Atlantic the kelp species *Saccharina latissima* (Laminariales, Phaeophyceae), a close relative of the commercially important *Saccharina japonica*, is especially interesting for cultivation purposes. This alga has an alternating life cycle during which large, multicellular sporophytes alternate with microscopic gametophytes (Fig. 1). From a selective breeding perspective, *S. latissima* has several advantages over other exploitable seaweeds that include the following: (1) full control over its life cycle, (2) both male and female gametophytes can be isolated and vegetatively propagated,^5^ and (3) selection and breeding can be performed in both the gametophytic and sporophytic phases.^6,7^ Selective breeding and intensive selection programs have been successfully used in China for improving disease resistance, growth rates, tolerance to high irradiance and water temperature.^8–10^ However, several Asian cultivars suffer from inbreeding depression, due to continuous “selfing” and limited crossbreeds from a restricted germplasm base.^11^

There is a general trend of decreasing abundance of several native kelp species, including *S. latissima*, at their southern distributional range limits and increasing abundance in other parts of their distribution, mainly northward.^4,12,13^ The founder effect of postglacial colonization in northern *S. latissima* populations suggests a lower intraspecific genetic diversity compared with populations from the southern range of distribution.^14,15^ Intraspecific genetic variability is critical for species adaptation and evolution, but diversity hotspots and refugia of kelp forests at the southern range of its distribution in the North Atlantic are predicted to disappear due to increasing seawater temperatures.^16^ Moreover, *S. latissima* kelp forests have been replaced by communities of opportunistic filamentous algae in sheltered and inner parts of the Norwegian Skagerrak,^17^ and a similar reduction has been observed in Sweden,^18^ Denmark, and Germany.^19^ Therefore, it would appear that not only southern range edge populations but also nontrailing edge populations might be vulnerable.^20^ To safeguard the existing intraspecies diversity for the future of the global seaweed aquaculture industry, national seed banks need to be established, thereby maintaining seed stock of developed strains and the intraspecific genetic resources.^21^

Cryopreservation offers the possibility for long-term, stable storage of living cells at ultralow cryogenic temperatures (generally lower than −120°C). Compared with current kelp seed banks, which use liquid media in which cultures actively grow,^5,22^ cryopreservation has several advantages; for example, the cells are less prone to pathogens and somatic mutations, and there are lower costs in terms of staff time and facilities. Cryopreservation has been successfully employed to conserve cyanobacteria and eukaryotic microalgae,^23–27^ with varying degrees of success in the conservation of macroalgae^23,28^ and kelp gametophytes.^22,29–32^ However, to date no study has explored the possibility of cryoconserving *S. latissima* or evaluated post-thawing recovery, sexual reproduction, and sporophyte development.

This study explores the use of different cryopreservation techniques on male and female *S. latissima* gametophytes originating from the Swedish west coast. Postcryopreservation viability, regeneration, and sporophyte development after thawing were assessed. The overarching aim of this project is to develop a method for the long-term preservation of living material of *S. latissima* through cryopreservation to facilitate the development of a future biobank capable of conserving commercially interesting strains, acting as a resource for future breeding or other experimental purposes, and the genetic resource management of wild populations.

## Materials and Methods

### Biological material

*S. latissima* sporophytes were collected on the Swedish west coast (58°83′ N, 10°99′ W). Mature sorus tissue (i.e., structure on the thallus consisting of clusters of sporangia containing and producing zoospores) was induced during a 10-week culture period in 10°C, short daylight period (8-hours light) with removal of the meristem (10–15 cm above the base).^33^ Sori were cleaned, and zoospores were released from each individual sporophyte into sterile seawater before being transferred to Petri dishes containing half strength Provasoli's enriched seawater (PES) medium and maintained at 10°C under 15 μmol photons m^−2^ s^−1^ red light, 16:8 hours light:dark cycle. Gametophytes were allowed to develop vegetatively for 2 months, after which male and female gametophyte colonies derived from two *S. latissima* individuals were isolated and transferred to 5 mL well plates. The gametophyte biomass was increased clonally by fragmentation, maintained under the same culture conditions, and the PES medium changed biweekly. For a detailed procedure on gametophyte clonal stock cultures, see Bartsch 2018. Clonal material was used to minimize variation in response to cryopreservation due to genotypic variation. Although standard aseptic techniques were used, the cultures were not axenic as the spores were released from cleaned but nonaxenic sorus tissue.^5^ Therefore, the effect of an antibiotic mix to prevent post-thaw bacterial growth was tested in a subsection of the samples. Penicillin G (1 g) and streptomycin (0.5 g) were dissolved in 90 mL of water, and chloramphenicol (0.1 g) was dissolved in 10 mL of 100% ethanol. This was mixed, filtered through a 0.22 μm membrane, and stored at −20°C. Finally, 5 mL of the antibiotic mix was added to 1 L of PES culture medium.

### Cryopreservation procedures

**FIG. 2.**
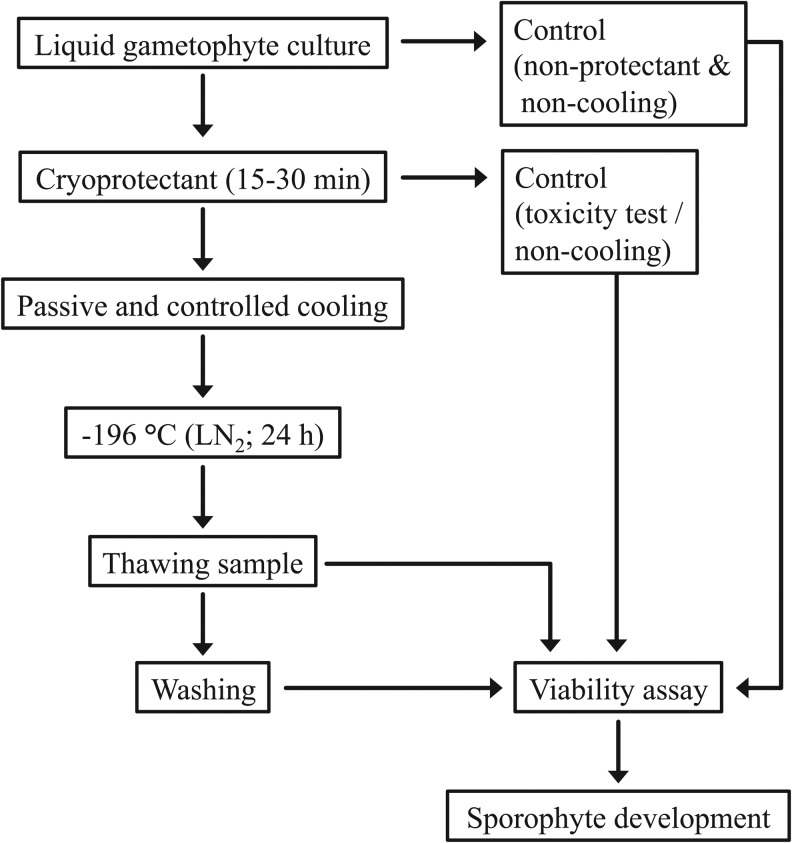
Procedure for the cryopreservation of *S. latissima* gametophytes and the viability assay.

The general workflow of the cryopreservation procedure and the viability assay used is shown in Figure 2, and was partially based on Heesch et al. More specifically, the cryoprotectants dimethyl sulfoxide (DMSO) (5% v/v), D-sorbitol (9% v/v) together with DMSO (10% v/v), polyethylene glycol (10% v/v), methanol (10% v/v), and polyethylene glycol (5% v/v) along with methanol (5% v/v) were used as cryoprotectants.^23,29,30,34,35^ They were dissolved in filtered autoclaved (15 minutes, 121°C) seawater at twice the final cryoprotectant concentration. D-sorbitol was added to natural, filtered seawater to a final concentration of 20% (w/v) before autoclaving (15 minutes, 121°C). After cooling to room temperature, DMSO (10 mL) was added to 90 mL of the sterile D-sorbitol solution, and polyethylene glycol (10 mL) and methanol (10 mL) were added to 90 mL of sterile seawater, resulting in the final concentration of cryoprotectant agents. Aliquots (10 mL) of the cryoprotectant solution were then filter-sterilized into sterile universal tubes, chilled, and aseptically dispensed in 1 mL aliquots into sterile cryogenic vials (2 mL capacity; Greiner bio-one). The cryogenic vials were cooled to 10°C before whole *S. latissima* gametophyte fragment colonies were transferred into the vials. After 15–30 minutes incubation under ambient light conditions, the samples were subjected to the different controlled-rate cooling methods tested. In addition, viability was evaluated after direct plunging the samples with and without the different cryoprotectants into liquid nitrogen.

### Controlled-rate freezing protocol

Two controlled-rate coolers were employed: (1) a controlled-rate cooler (Planer plc, KRYO 360–3.3) and (2) a “nitrogen-free” Stirling cycle freezer (Asymptote EF600; Cell Cryogenics Ltd.). In the controlled-rate cooler, ice nucleation was induced in the samples (replicated three times [*n* = 3]) in a controlled manner when cooled. Cooling started at +10°C at 1°C min^−1^ to −40°C, holding for 10 minutes at −40°C before the samples were rapidly transferred to a small dewar containing liquid nitrogen (−196°C). In the Stirling cycle freezer, ice nucleation in the samples was induced in a controlled manner by means of the “plate technology” in the freezer that provides even cooling across all samples. Cooling started at +10°C at 1°C min^−1^ to −80°C with a 15-minute intermediate stationary phase at −40°C before the samples were transferred to liquid nitrogen vapor phase ultracold storage (less than −150°C). Samples were stored for 24–28 hours before further manipulations.

### Passive rate freezing protocol

Samples were loaded onto two low-tech passive rate coolers in which ice nucleation occurs spontaneously; Mr. Frosty^®^ freezing container (ThermoFisher Scientific) and CoolCell^®^ freezing container (FTS30; BioCision) are placed into a conventional −80°C freezer. In both passive rate cooling methods, the samples were cooled to −80°C at a nonlinear rate of <1°C min^−1^, after which the samples were immediately immersed in liquid nitrogen or its vapor phase (less than −150°C) and stored for 24–28 hours. The replication and cryopreservation procedures were identical as described above with the exception that replication in the Mr. Frosty method was in duplicate (*n* = 2), due to lower vial capacity.

### Recovery procedure and viability assessment

After storage in liquid nitrogen, the vials were rapidly warmed in a water bath at +40°C and transferred to a laminar-flow cabinet immediately after melting of all the ice. Samples were removed from the vials, transferred to a Petri dish, washed with 10 mL of fresh PES medium using standard aseptic techniques, and incubated in the dark at 8°C for 1 day. Thereafter, the samples were transferred to six-well plates (12 mL) and washed with fresh medium before exposure to standard culture conditions (PES medium 15 μmol photons m^−2^ s^−1^ [PAR] 14L:10D at 10°C). Medium was changed weekly, and additional washing steps were performed if bacterial growth was observed.

Viability of the gametophytic cells was assessed at days 10, 24, and 35 post-thawing on the basis of five levels of culture viability (no viability; 1%–20% viability; 20%–50% viability; 50%–80% viability; and >80% viability) by light microscope (Leica Labovert inverted microscope or Zeiss Axiovert 200). Culture viability was visually estimated as the proportion of brown colored cells (i.e., viable cells) of the total number of gametophytic cells within a sample. For example, if 1%–20% viability was reported at day 10, as well as on day 24 and 35 post-thawing, the number of viable cells increased, but due to the slow growth, viability did not exceed 20% of the initial cryopreserved cells. The effects of antibiotic treatment on gametophyte viability were assessed similarly after 8, 16, and 30 days. Sporophyte development was assessed by crossings with noncryopreserved male or female gametophytes as appropriate (mix of six different individuals) at day 35 except for treatments with very low viability (i.e., passive rate freezing protocols) that were crossed 52 days post-thawing (Supplementary Table S1). This was done to allow samples with low cell viability to vegetatively (i.e., clonally) propagate more gametophytic cells, thereby increasing the possibility to develop into sporophytes. The presence of sporophytes was analyzed 16 days postcrossing.

## Results

### Effect of cooling method on survival of cryopreserved gametophytes

**FIG. 3.**
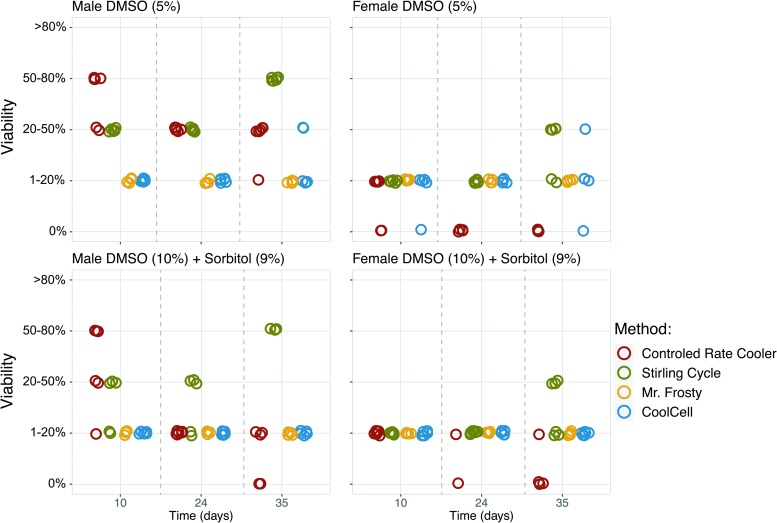
Changes in viability and recovery of male and female *S. latissima* gametophytic cells during post-thawing incubation for all four cooling methods and two protectants (*n* = 6, n_Mr. Frosty_ = 4). Color images are available online.

**FIG. 4.**
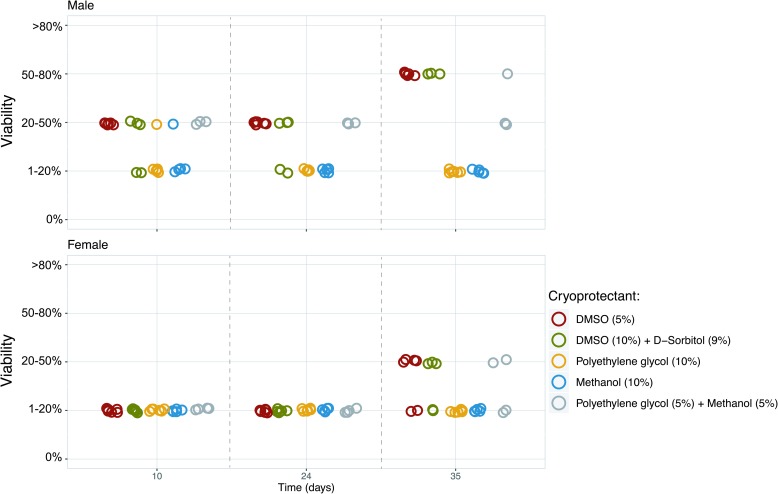
Changes in viability of male and female *S. latissima* gametophytic cells during post-thawing incubation using the Stirling cycle freezer method in combination with the different cryoprotectants (*n* = 6, n_Polyethylene glycol (5%)+Methanol (5%)_ = 4). Color images are available online.

**FIG. 5.**
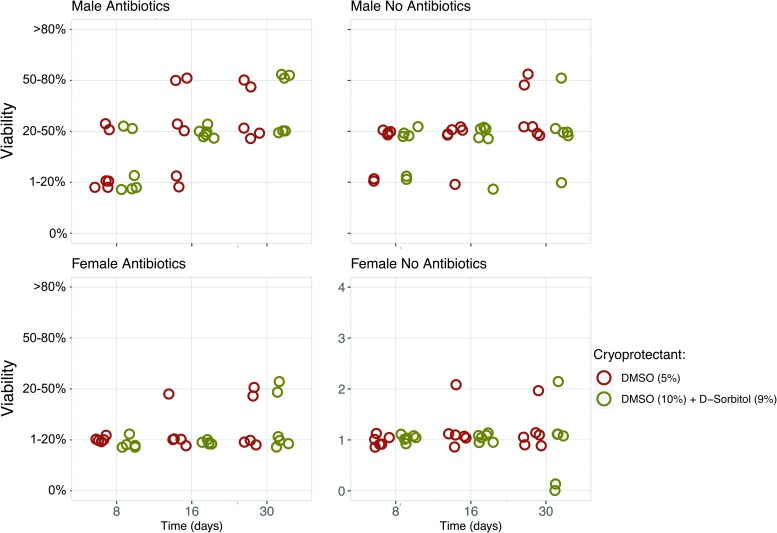
Effect of precryopreservation antibiotic treatment on changes in viability during post-thawing incubation of male and female *S. latissima* gametophytic cells after cryopreservation using the Stirling cycle freezer method combined with two cryoprotectants (*n* = 6). Color images are available online.

The effect of cooling method on the survival rate of gametophytes of *S. latissima* after 10 days is shown in Supplementary Table S2 and Figure 3. All cooling methods resulted in viable gametophytic cells after thawing, but no viability was detected when gametophytes were plunged directly in liquid nitrogen (−196°C). For all protocols employed, extracellular ice nucleation occurred during the first cooling phase. Overall, the controlled-rate freezing methods (controlled-rate cooler and Stirling cycle freezer) showed higher viability compared with the passive rate freezing methods (Mr. Frosty and CoolCell), although this difference was mainly observed for male gametophytes. No difference was observed between the two passive cooling methods, nor between the two controlled cooling methods. In addition, higher viability was noted in male gametophytes than in female gametophytes (Figs. 3–5).

### Effect of cryoprotectants on survival of cryopreserved gametophytes

The effects of cryoprotectants on the survival rate of gametophytes of *S. latissima* are reported in Table 1 and Figure 4. No toxicity effect of the cryoprotectants on gametophyte viability was observed. After cooling, signs of surviving gametophytic cells were noted for all cryoprotectants, with higher survival levels when using the cryoprotectants DMSO (5%) and DMSO (10%)+D-sorbitol (9%), compared with when polyethylene glycol (10%) and methanol (10%) were employed. Again, this difference was mainly observed for male gametophytes.

**FIG. 6.**
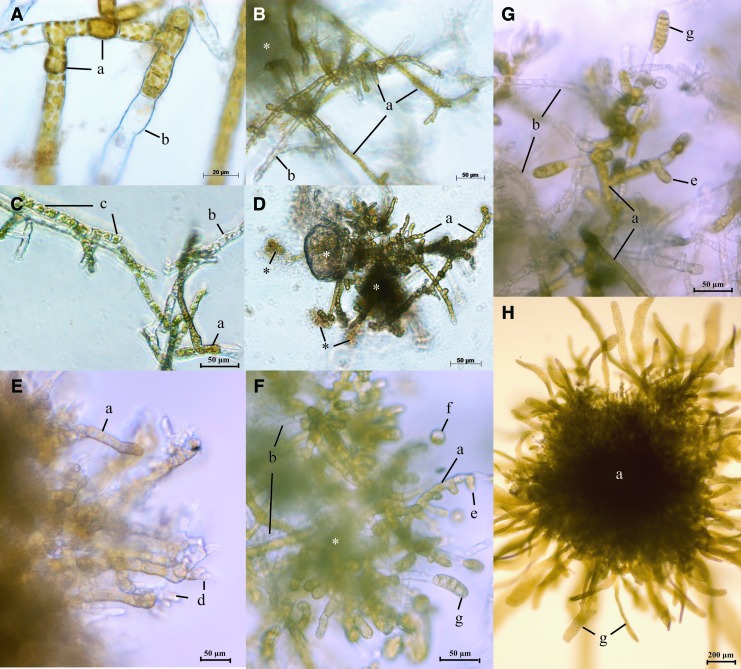
Recovery and development of cryopreserved male and female gametophytic cells. **(A)** Female gametophyte 2 days post-thawing, the viable cells contain intact plastids with brown pigmentation; **(B, C)** Male gametophyte 2 days post-thawing with brown pigmented viable cells, plasmolyzed and flaccid cells, and empty transparent lysed cells; **(D)** Unwashed male gametophyte 10 days post-thawing showing viable cells affected by the anaerobic environment created by bacteria growing on the cryoprotectant and released cell content of lysed gametophytic cells; **(E)** Recovered male gametophytic cells 42 days post-thawing with antheridia formed at the tip of its filaments; **(F–H)** Fully recovered female gametophytic cells showing gametogenesis and the formation of morphological normal sporophytes after fertilization by noncryopreserved control male gametophytes 60 days post-thawing. a, viable gametophytic cell; b, lysed gametophytic cell; c, plasmolyzed and/or flaccid gametophytic cell; d, antheridia; e, oogonia; f, egg; g, sporophyte; *, bacterial growth and cryoprotectant. Color images are available online.

Washing off the cryoprotectant had a significant effect on long-term (>10 days post-thawing) gametophyte viability. Samples cryopreserved using the controlled-rate cooler method were only washed once, directly after thawing, resulting in high bacterial contamination after 10 days post-thawing (Fig. 6D). The bacterial contamination in these samples was accompanied with a decrease in gametophyte viability observed 24 and 35 days post-thawing (Supplementary Tables S3 and S4), compared with the Stirling cycle treatment. This was most notable when DMSO (10%) with sorbitol (9%) was used (Fig. 3).

Addition of the antibiotic mix precryopreservation prevented bacterial growth, and resulted in an increased long-term recovery and vegetative clonal growth (i.e., active cell division) similar to prior preservation (Fig. 5). When bacteria were present in the sample, the gametophytes were totally covered, which negatively influenced growth and specifically its recovery (Fig. 3, controlled-rate cooler method).

### Sporophyte development of cryopreserved gametophytes

The development of male and female reproductive organs, antheridia and oogonia (eggs), respectively (Fig. 6E–G), generated by the cryopreserved gametophytes was directly related to post-thawing viability. No effect of the preservation method or cryoprotectant on the development and morphology of sporophytes was observed (Fig. 6G, H). However, bacterial growth halted the formation of new clonal gametophytic cells and reproductive structures, preventing sporophyte formation in these samples.

## Discussion

In this study, we successfully developed a standardized and widely applicable two-step cooling protocol suitable for the long-term stable preservation of kelp gametophytes. However, despite a limited genotypic variation among samples, the viability of gametophytic cells varied among the tested freezing protocols. The highest viability levels observed after cryopreservation were in samples using a controlled-rate freezing method (i.e., controlled-rate cooler and Stirling cycle freezer methods) in combination with cryoprotectant DMSO (5%) or DMSO (10%) + D-Sorbitol (9%). This difference was mainly noted for male gametophytes. The passive cooling methods (Mr. Forsty and CoolCell) resulted in lower viability levels. This finding was reflected in the number of developing sporophytes after crossing of cryopreserved male and female gametophytes with their noncryopreserved counterparts among all treatments. We also noted higher viability in samples of male gametophytes compared with female gametophytes after cryopreservation. In addition, we show for the first time that the N_2_-free Stirling cycle freezer technique can be successfully applied in the cryopreservation of kelp gametophytic types of cells (i.e., multicellular filamentous algal cells), in accordance with what has been reported earlier for other cell types.^36–40^ This finding is interesting since the Stirling Engine is a closed cycle unit in which the refrigerant working fluid is contained inside the machine, and only a source of mechanical or electrical energy is required to reach temperature below −100°C,^41^ thus allowing the process to be performed where liquid nitrogen is not available, as stored samples may be held in an ultracold electrically powered freezer at temperatures less than −120°C.

The viability levels, of up to 80% for male gametophytes and 20% for female, reported in this study are broadly comparable with previously reported survival levels (between 42% and 100%) obtained using a similar two-step cooling protocol for other brown algae gametophytes.^29,32,34,35,42,43^ There are other cryopreservation protocols available, but they have generally resulted in lower viability when compared with traditional two-step cooling protocols. For example, cryopreservation by encapsulation–vitrification of *Undaria pinnatifida* gametophytes resulted in viabilities between 26% and 31%,^44^ and survival rates of up to 43% were observed when an encapsulation–dehydration protocol was applied to *Laminaria japonica* gametophytes.^31^ Despite the relatively low female viability reported in this study, post-thawing crossings have resulted in morphological normal sporophytes, indicating that low viability is sufficient for the development of sporophytes. However, future research into protocol optimization is needed, especially for female gametophytes.

Similar to our results, higher male survival compared with female gametophytic cells has also been found in other brown algae gametophytes.^31,32,42,43^ It has been speculated that this might be a result of morphological differences between sexes, with slender and longer male gametophytic cells compared with thicker and larger female gametophytic cells,^45^ possibly allowing the cryoprotectant to penetrate into the male cells more easily whereby it loses more water than big cells during freezing, preventing intercellular ice nucleation.^46^ When preserving female cells, applying different treatment lengths then for male cells (e.g., longer), might allow the cryoprotectants to penetrate better into the female cells, thereby possibly enhancing viability. Our results also confirm that plunging gametophytes directly into liquid nitrogen, without applying any cryoprotectant or initial controlled-rate cooling to prevent cell injury by intracellular ice formation, does not result in the generation of viable gametophytic cells.

The estimation of cell survival by morphological and coloration examination of the cryopreserved gametophytic cells using light microscopy was shown to be a reliable method for this alga, as the estimated viability post-thawing was a good indicator of the total number of developed sporophytes per treatment. Viable gametophytic cells had a brown color, strongly contrasting with discolored lysed cells that were not viable (Fig. 6). However, cells with a slight discoloration, that is, from brown to light brown/yellow and less strongly contrast with lysed cells can also be the result of plasmolysis (Fig. 6C). As reported by Ginsburger-Vogel et al.,^47^ freezing injuries at the cellular level can cause alterations in the cell structure, probably due to cytotoxic leakage of vacuolar content to the cell cytoplasm, and modifications in the arrangement of thylakoids and discoloration of the cells.

Cryoinjuries and stresses associated with the cryopreservation protocol applied have to be addressed to enhance postcryopreservation viability.^48^ A number of strategies for improving post-thaw viability of cryopreserved cells have been reported, most of them involving manipulations of precryopreservation culture conditions.^32,47,49,50–52^ We tested an antibiotic mix treatment 24 hours precryopreservation, and observed positive effects on recovery and vegetative clonal cell division of cryopreserved gametophytic cells. A combination of factors such as insufficient removal of the cryoprotectant from the sample and additional input due to cell content of lysed cells as a result of cryoinjuries might lead to the excessive bacterial growth. Together with the remaining cryoprotectant, the bacteria form a biofilm around the gametophytes leading to unfavorable conditions and ultimately dead cells (Fig. 6D). In this study, we observed a higher post-thaw viability when the Stirling cycle cooling method was used compared with the controlled-rate cooler method. The latter method, containing less washing after thawing, was associated with high bacterial contamination after 10 days post-thawing that was most likely the cause of the lower viability of gametophytes.

Adjustments to the applied cooling protocol that might improve post-thawing cell viability also involve the cooling rate (e.g., passive or controlled), holding time, holding temperature, and their relative interactions.^42^ Especially cryoprotectant type and incubation time are both important to consider due to the cell wall, preventing some types of cryoprotectants from penetrating into the cell.^29,51^ To achieve high viability not only must intracellular free water be removed, but too intense dehydration should also be avoided. This study focused on penetrating cryoprotectants (e.g., DMSO and glycol) that act colligatively, but may produce temporary plasmolysis as they penetrate the wall and loosen adhesion between the wall and cell membrane (e.g., Fig. 6C). Toxicity of the cryoprotectant may also lethally injure cells, but no toxic effect of the cryoprotectants used in this study was found on *S. latissima* gametophytic cells. Previous studies have demonstrated that a combination of cryoprotectant classes may result in the highest survival levels,^23,53^ as also shown in this study where increased male viability was observed when polyethylene glycol was combined with methanol (Fig. 4). A future strategy to improve protocols for cryopreserving kelp gametophytes and achieve higher reliability might be to use a cocktail of different cryoprotectants.

The success of different types of cryopreservation strategies for the long-term storage of brown algae gametophytes is clearly dependent upon the susceptibility of the cell type to cryoinjury as a result of ultralow temperatures, extracellular freezing, and osmotic stress. The initial controlled-rate cooling phase, cryoprotectant, and post-thawing washing phase had a profound influence on the gametophytes' capacity to survive these stresses, recover, and ultimately transfer into the sporophytic life stage. In other marine algal species, viability after short-term cold storage (e.g., 24 hours) at ultralow temperatures is highly correlated with viability after long-term storage (e.g., month to years).^54–56^ However, long-term storage of marine microalgae at cryogenic temperatures (−196°C) has resulted in some alterations in physiological responses, although no reduction in viability was observed.^57^ Furthermore, long-term storage at cryogenic temperatures may cause molecular alterations^36,58^ and effects on gametophyte genome integrity and its effect on sporophyte development would be an interesting next step in future cryopreservation studies.

In conclusion, *S. latissima* male and female gametophyte clones were successfully conserved using a two-step cryopreservation protocol. The controlled-rate cooling methods generated higher viability than the low-tech passive cooling methods, but both methods resulted in viable gametophytic cells with the ability to successfully complete its life cycle. Thus, the methods for the long-term preservation of living material of *S. latissima* evaluated here have potential to facilitate the development of a future biobank capable of conserving commercially interesting strains, thereby safeguarding the future seaweed aquaculture industry, and act as a resource for future breeding or other experimental purposes or the genetic diversity of wild populations.

## Supplementary Material

Supplemental data

Supplemental data
