# Supplementary material for: Underpinning the Development of Seaweed Biotechnology: Cryopreservation of Brown Algae (Saccharina latissima) Gametophytes
Source: Biopreserv Biobank. 2019 Oct 10;17(5):378–86. doi: 10.1089/bio.2018.0147 (PMC6791476; doi:10.1089/bio.2018.0147)
Supplement: Supplemental data [file Supp_Table1-2.pdf]

Supplementary Data

Supplementary Table S1. Effects of Various Cryoprotectants and Preservation Methods on the Viability of Male (♂) and Female (♀) Gametophytic Cells of *Saccharina latissima* 52 Days Post-Thawing (Median, $N=6$ and $N_{Mr.\ Frosty}=4$)

	Survival											
	Cryoprotectant (noncooling)		*LN2 (direct plunge)*		*Controlled rate cooler*		*Stirling cycle freezer*		*Mr. Frosty*		*CoolCell*	
Cryoprotectant	♂	♀	♂	♀	♂	♀	♂	♀	♂	♀	♂	♀
5% (v/v) DMSO	Crossed*		− − −	− − −	Crossed*		Crossed*		Crossed*		Crossed*	
10% (v/v) DMSO +9% (w/v) D-sorbitol			− − −	− − −								
10% (v/v) polyethylene glycol			− − −	− − −			+ + +	− − +	+ + +	− − +	+ + +	− − +
10% (v/v) methanol			− − −	− − −			− − +	− − −	− − +	− − −	− − −	− − −
5% (v/v) polyethylene glycol +5% (v/v) methanol			− − −	− − −			Crossed*					

− − − = no viability.
− − + = 0–20% viability.
− + + = 20–50% viability.
+ + + = 50–80% viability.
+ = >80% viability.
*Crossed, start induction of gametogenesis and fertilization 35 days post-thawing.

Supplementary Table S2. Effects of Various Cryoprotectants and Preservation Methods on the Viability of Male (♂) and Female (♀) Gametophytic Cells of *Saccharina latissima* 10 Days Post-Thawing (Median, $N=6$)

	Survival											
	Cryoprotectant (noncooling)		*LN2 (direct plunge)*		*Controlled rate cooler*		*Stirling cycle freezer*		*Mr. Frosty*		*CoolCell*	
Cryoprotectant	♂	♀	♂	♀	♂	♀	♂	♀	♂	♀	♂	♀
5% (v/v) DMSO	+	+	− − −	− − −	+ + +	− − +	− + +	− − +	− − +	− − +	− − +	− − +
10% (v/v) DMSO +9% (w/v) D-sorbitol	+	+	− − −	− − −	+ + +	− − +	− + +	− − +	− − +	− − +	− − +	− − +
10% (v/v) polyethylene glycol	+	+	− − −	− − −			− − +	− − +	− − +	− − +	− − +	− − +
10% (v/v) methanol	+	+	− − −	− − −			− − +	− − +	− − +	− − +	− − +	− − +
10% (v/v) polyethylene glycol +10% (v/v) methanol	+	+	− − −	− − −			− + +	− − +				

− − − = no viability.
− − + = 0–20% viability.
− + + = 20–50% viability.
+ + + = 50–80% viability.
+ = >80% viability.
DMSO, dimethyl sulfoxide.
